# Integrative genetic analysis suggests that skin color modifies the genetic architecture of melanoma

**DOI:** 10.1371/journal.pone.0185730

**Published:** 2017-10-03

**Authors:** Imge Hulur, Andrew D. Skol, Eric R. Gamazon, Nancy J. Cox, Kenan Onel

**Affiliations:** 1 Committee on Genetics, Genomics and Systems Biology, The University of Chicago, Chicago, Illinois, United States of America; 2 Department of Medicine, The University of Chicago, Chicago, Illinois, United States of America; 3 Department of Pediatrics, Hofstra Northwell School of Medicine, Hempstead, New York, United States of America; 4 Department of Genetics and Genomics, The Feinstein Institute for Medical Research, Manhasset, New York, United States of America; University of Arizona, UNITED STATES

## Abstract

Melanoma is the deadliest form of skin cancer and presents a significant health care burden in many countries. In addition to ultraviolet radiation in sunlight, the main causal factor for melanoma, genetic factors also play an important role in melanoma susceptibility. Although genome-wide association studies have identified many single nucleotide polymorphisms associated with melanoma, little is known about the proportion of disease risk attributable to these loci and their distribution throughout the genome. Here, we investigated the genetic architecture of melanoma in 1,888 cases and 990 controls of European non-Hispanic ancestry. We estimated the overall narrow-sense heritability of melanoma to be 0.18 (*P* < 0.03), indicating that genetics contributes significantly to the risk of sporadically-occurring melanoma. We then demonstrated that only a small proportion of this risk is attributable to known risk variants, suggesting that much remains unknown of the role of genetics in melanoma. To investigate further the genetic architecture of melanoma, we partitioned the heritability by chromosome, minor allele frequency, and functional annotations. We showed that common genetic variation contributes significantly to melanoma risk, with a risk model defined by a handful of genomic regions rather than many risk loci distributed throughout the genome. We also demonstrated that variants affecting gene expression in skin account for a significant proportion of the heritability, and are enriched among melanoma risk loci. Finally, by incorporating skin color into our analyses, we observed both a shift in significance for melanoma-associated loci and an enrichment of expression quantitative trait loci among melanoma susceptibility variants. These findings suggest that skin color may be an important modifier of melanoma risk. We speculate that incorporating skin color and other non-genetic factors into genetic studies may allow for an improved understanding of melanoma susceptibility and guide future investigations to identify melanoma risk genes.

## Introduction

With 76,380 new cases of invasive melanoma and 68,480 new cases of melanoma *in situ* expected in 2016 [1], melanoma incidence is rising more rapidly than that of any other cancer in the United States [2–5]. Although melanoma accounts for only 1% all skin cancer cases, it is responsible for the majority of skin cancer deaths [1]. In addition to its high mortality, melanoma is associated with enormous health care costs. The annual cost of productivity loss and direct medical care associated with melanoma in the United States is estimated at $3.5 billion and $932.5 million annually, respectively [6, 7].

Melanoma primarily affects individuals of European ancestry and is much less common among individuals of Asian, African and Hispanic ancestry [8]. The main environmental risk factor for melanoma in whites is ultraviolet radiation (UVR) from sunlight, which promotes cancer by multiple mechanisms including direct DNA damage [9, 10], immunosuppression [11, 12], and dysregulation of growth factors [13]. Another major risk factor for melanoma is light skin color, which is characterized by low levels of the UVR-absorbing pigment melanin [14]. Even in white individuals, differences in melanin are associated with differences in the amount of UVR to which melanocytes are exposed. Consequently, light-skinned whites are two to three times more likely to develop melanoma than are dark-skinned whites [15, 16].

Genetics also plays an important role in melanoma susceptibility [17]. Genome-wide association studies (GWAS) have identified several single nucleotide polymorphisms (SNPs) associated with melanoma risk, many of which many influence pigmentation [18–25]. GWAS have also discovered melanoma susceptibility loci that are not associated with pigmentation; many of these are thought to mediate the response to UVR damage by regulating genes involved in DNA repair and cell cycle control [25, 26].

There have been a few studies that aim to characterize the proportion of melanoma risk attributable to genetics. The heritability of melanoma was estimated to be 55% in a study of Australian twins [27], and 58% in a study of Nordic twins [28]. More recently, a software tool called Genome-wide Complex Trait Analysis (GCTA), which allows for the estimation of heritability using genome-wide genotype data from unrelated individuals [29], was applied to quantify the contribution of genetic factors to melanoma susceptibility. In this study, heritability of melanoma was estimated to be 19% and 30% based on two different melanoma GWAS datasets from the USA and Australia, respectively [30].

In this study, we utilized an integrative approach, intersecting genome-wide genotype data from a melanoma GWAS with heritability analyses using expression quantitative trait loci (eQTL) and melanoma-associated covariates to gain insights into melanoma susceptibility, including the relative contributions of genetic factors and the underlying genetic architecture and functional mechanisms. For our analyses, we used the USA GWAS dataset mentioned in the previous paragraph, which had yielded the GCTA heritability estimate of 0.19 [30]. Here, we partitioned that heritability by chromosome, minor allele frequency (MAF) and functional annotation, including genic regions and skin *cis*-eQTLs, in an attempt to characterize the genomic distribution and allelic frequency spectrum of melanoma risk variants and to determine if any particular class of variation is driving melanoma risk. Given that pigmentation is a known melanoma risk factor, we incorporated skin color into our heritability analysis and performed a GWAS conditional on skin color. In addition to identifying risk loci that reached genome-wide significance, we also found evidence that these variants were enriched for skin *cis*-eQTLs, highlighting the importance of genetic regulation of gene expression in melanoma susceptibility.

Taken together, these results underscore the importance of incorporating the influence of non-genetic factors such as skin color into genetic analyses of complex traits such as melanoma, as they can significantly modify the underlying genetic architecture.

## Methods

### Melanoma data set

We obtained data from the Gene Environment Association Studies (GENEVA) melanoma project GWAS available through Database of Genotypes and Phenotypes (dbGaP) (accession number phs000187.v1.p1) [31]. Details of the case and control samples and genotyping have already been published [21]. Briefly, the dataset included 1,971 cases and 1,045 controls of European descent. All cases and controls were genotyped using the Illumina HumanOmni1-Quad (v1.0) array, and 1,012,904 satisfactorily genotyped SNPs were released to dbGaP. Skin color information was available for all controls and approximately half the cases (N = 1,050). Skin color was self-reported, obtained via questionnaire in which subjects were asked to grade their skin color on a scale of 1 (very fair) to 10 (dark brown).

### SNP genotype quality control (QC)

#### Standard QC

We excluded SNPs with MAF < 0.02, missingness > 2%, and Hardy Weinberg equilibrium (HWE) test *P* ≤ 10^−4^. Sex chromosomes and insertions/deletions (indels) were excluded from the analysis. We excluded samples with a genotype call rate ≤ 95%. SNP chromosomal positions were converted from hg18 to hg19 coordinates using liftOver (http://genome.ucsc.edu/cgi-bin/hgLiftOver). After QC, we were left with 775,275 SNPs genotyped in 1,968 cases of melanoma and 1,044 controls with a mean sample call rate of 99.93%.

#### QC for heritability estimates

We used more stringent QC criteria when filtering SNPs and individuals to estimate heritability, as recommended by Lee et al (2011) [32], in order to minimize artifactual differences between case and control allele frequencies, because not doing so can artificially inflate heritability estimates. We excluded SNPs with MAF < 0.01, missingness > 5%, and HWE *P* < 0.05. We further excluded SNPs with *P* < 0.05 for tests of differential missingness between cases and controls. We restricted our analysis to autosomal SNPs. We excluded cases with genotype call rate ≤ 99%, or an estimated genetic relatedness score (by GCTA) of > 0.05. After the stringent QC process, we were left with 1,888 cases and 990 controls, and 756,002 SNPs from which to estimate heritability.

### Principal components analysis (PCA)

We investigated the population structure of our dataset by performing a PCA-based analysis using EIGENSTRAT [33] and including the European (CEU), African (YRI) and Asian (ASN = CHB + JPT) HapMap samples (S1 Fig). 33 individuals were identified as having non-European ancestry and excluded from the association and heritability analyses. Upon the removal of population outliers, we repeated the PCA, with CEU, Tuscan (TSI) and Ashkenazi Jewish (AJ) samples included as references and the GWAS samples color-coded according to case-control status (S2 Fig). The majority of samples clustered with the CEU, while a small number clustered with the AJ or trailed towards the TSI, suggesting that our study population is mostly of Northern European ancestry. We conducted the PCA without the reference population samples and used the first two PCs from the analysis to adjust for confounding by population stratification in the association analyses (S3 Fig).

### Imputation

We imputed 1000 Genomes Phase I SNPs with IMPUTE2 v2.2.2 using the 1000 Genomes Phase I integrated variant set release (v3) [34, 35]. We converted imputation probabilities into genotypes for all SNPs with info scores > 0.30 via thresholding, using a posterior genotype probability threshold of 0.90, as implemented in GTOOL v0.7.5 (http://www.well.ox.ac.uk/~cfreeman/software/gwas/gtool.html). Imputed SNPs with MAF < 0.01, missingness rate > 5%, HWE *P* ≤ 10^−4^ and all indels were excluded. Three samples with missingness rate > 5% for the imputed SNPs were removed, resulting in a final dataset of 6,311,517 SNPs in 1,952 cases and 1,025 controls.

### Genome-wide association study

We performed a melanoma GWAS using directly genotyped and imputed SNPs for 1,968 cases of melanoma and 1,044 controls. Specifically, we performed logistic regression on case–control status using the SNP genotype (additive effect), age, sex, and the first two PCs as covariates. (S3 Fig). We also performed a second GWAS in which skin color (coded as 1–10) was added as a quantitative covariate in the model.

### Heritability analysis of melanoma

We estimated the heritability of melanoma risk using GCTA v1.24 [29]. We first constructed a genetic relatedness matrix (GRM) that contains estimates of genetic relatedness for all pairs of individuals based on genotyped data after stringent QC. The additive genetic relationship between individuals *j* and (*A*_*Jk*_) is calculated by the following equation:

$$A_{jk}=\frac{1}{N}\sum_{i=1}^{N}\frac{\left(x_{ij}-2p_{i}\right)\left(x_{ik}-2p_{i}\right)}{2p_{i}\left(1-p_{i}\right)}$$

where *N* is the number of SNPs, *x*_*ij*_ is the number of copies of the reference allele for the *i*^*th*^ SNP of the *j*^*th*^ individual and *p*_*i*_ is the frequency of the reference allele. GCTA then fits the GRM as random effect by a mixed linear model (MLM) to estimate the total additive variance explained by all SNPs ($\sigma_{g}^{2}$). The MLM is represented by the following equations:

$$\begin{array}{lll} y=X\beta +g+\varepsilon & \text{with} & var \end{array}\left(y\right)=V=A\sigma_{g}^{2}+I\sigma_{\varepsilon }^{2}$$

where *y* is a vector of phenotypes, *β* is a vector of fixed effects such as age, sex or ancestry principal components (PC), *g* is a vector of total additive genetic effects, *ε* is a vector of residual effects, *I* is an identity matrix, A is the GRM and $\sigma_{\varepsilon }^{2}$ is error variance.

Our strict QC measures substantially reduce the number of SNPs used for the GRM, which decreases linkage disequilibrium (LD) between the remaining SNPs and the true risk loci and leads to a downwardly biased estimate of heritability. We used the method described in Yang et al (2010) [36] to correct this bias.

PCs of the genotype data were calculated using GCTA. The top 20 PCs were included as quantitative covariates in the heritability analysis to account for any possible persistent population structure that might result in inflated heritability estimates. Case–control status was transformed to a liability scale via a probit model and fit as a mixed model that included the top 20 PCs as fixed effects and the additive genetic relatedness as a random effect. The additive genetic variance was estimated using restricted maximum likelihood (REML) in GCTA. A further adjustment to heritability is made to correct for the ascertainment bias that results due to the difference in the prevalence of melanoma in our sample (66%) relative to its population prevalence (2%).

### Heritability explained by known melanoma SNPs

We downloaded all SNPs previously identified in GWAS as associated with melanoma from the National Human Genome Research Institute (NHGRI) catalog (http://www.ebi.ac.uk/gwas), using the default p-value threshold of 1 x 10^−5^ (S1 Table). Out of the 39 variants, 17 were directly genotyped in our dataset and 16 were imputed but not directly genotyped. 6 SNPs were not genotyped or imputed in our study, and there was also no LD information regarding them in either the HapMap or 1000 Genomes Project databases. Hence, these SNPs were excluded from our analysis. We generated the GRM using the remaining 33 GWAS SNPs for which we had direct or imputed genotype data. The heritability attributable to these melanoma-associated SNPs was then estimated via the REML analysis described above. In addition, we repeated the heritability analysis including all SNPs within 250 kb (up and downstream) of the 39 known melanoma-associated SNPs (N = 5,324). In order to test the significance of the heritability estimate explained by known GWAS SNPs (or GWAS regions), we generated 1,000 permutations of the melanoma case-control status and reran GCTA on each permutation. The empirical p-value was estimated as the number of permutations in which the heritability exceeds the heritability estimated using the correct case-control assignments.

### Heritability partitioned by chromosome

We generated separate GRMs for each chromosome and fit them all simultaneously in a single joint analysis to calculate the heritability explained by each chromosome. By plotting the heritability of each chromosome by its length, we sought to see whether there was a linear relationship between chromosome length and heritability, as would be expected under a polygenic model, where thousands of risk variants are distributed evenly across the genome.

### Heritability partitioned by minor allele frequency

In order to characterize the allelic frequency spectrum of the SNPs contributing to melanoma risk and to test if there is a disproportionately high contribution to risk from a particular MAF class, we partitioned the genotyped SNPs into six non-overlapping MAF bins from 0.01–0.05, > 0.05–0.1, > 0.1–0.2, > 0.2–0.3, > 0.3–0.4, > 0.4–0.5 and calculated the GRM separately for each MAF bin. We fit them all simultaneously using GCTA to estimate the heritability attributable to each MAF bin.

### Rare variants model

We used the method described in Yang et al. (2010) [36] to calculate the heritability of melanoma under the assumption that causal variants have MAF ≤ 0.1. Briefly, we estimated the GRM using only the SNPs with MAF ≤ 0.1 (*G*_*jk*_) and calibrated the prediction error by the regression of *G*_*jk*_ on the GRM estimated using all genotyped SNPs (*A*_*jk*_). Using the equation $\beta =1-\frac{\left(c+1/N\right)}{var\left(A_{jk}\right)}$, where *β* is the regression coefficient and *N* is the number of SNPs used to calculate *A*_*jk*_, we estimated *c*, the correction term for adjusting the GRM. We used the—*grm-adj* option in GCTA to adjust the GRM (*A*_*jk*_) with the estimated value of *c* (6.2 x 10^−6^).

### Heritability partitioned by functional annotation

#### Genic

We partitioned the SNPs into genic and intergenic regions using ANNOVAR (hg19, refGene) annotations [37]. Genic variants included those located within exons, coding sites, 3’- and 5’- untranslated regions (UTRs), splice sites and within 1 kb of the transcription start or stop sites. A single GRM was created for each partition and the heritability explained by each was determined in a single joint analysis where both GRMs were fit together.

#### Expression QTL

In a second analysis, we partitioned variants into skin *cis*-eQTLs and non-eQTL SNPs based on data from the MuTHER study (Multiple Tissue Human Expression Resource– https://www.ebi.ac.uk/arrayexpress/experiments/E-TABM-1140) [38]. As 32% of the SNPs that were used to estimate heritability in our study were not analyzed in the MuTHER study, we extracted SNPs that were common to both studies (N = 514,445) and calculated the heritability attributable to this subset of SNPs only. A single GRM was created for each partition and the heritability explained by each was determined in a single joint model where both GRMs were fit together. The analysis was repeated at three different *cis*-eQTL significance thresholds (0.01, 0.001, 0.0001).

### eQTL enrichment analysis

We conducted a test to determine if melanoma-associated SNPs were enriched for eQTLs using a permutation-based analysis described previously by Gamazon et al (2013) [39]. Specifically, we performed 1,000 GWAS under the null hypothesis of no association by shuffling case-control status while keeping the genotype data fixed to preserve the LD structure. In each permuted dataset, we determined the number of skin *cis*-eQTLs (defined as either *P* < 0.01 or *P* < 0.001) among the top 1,000 and 2,000 most significant GWAS SNPs. The empirical p-value for eQTL enrichment among the melanoma-associated SNPs is the proportion of permutations with more skin *cis*-eQTL observed in the top melanoma-associated SNPs than in our original GWAS. We conducted the enrichment analysis twice, once with and once without using skin color as a covariate in the GWAS.

## Results

### Association analysis

Following QC and imputation, we had genotype data on 6,311,517 SNPs in 1,952 cases and 1,025 controls of European descent from the GENEVA melanoma project. Using logistic regression, we successfully replicated the findings previously reported for this dataset [21]. These findings included SNPs in the *MC1R* region on chromosome 16 which surpassed the threshold for genome-wide significance (*P* = 1.4 x 10^−9^ for the index SNP rs74800773) in both our analysis and previously published analysis; rs1129038 tagging the *HERC2/OCA2* region on chromosome 15 (*P* = 3.1 x 10^−7^) which reached genome-wide significance in previously published analysis but only suggestive levels of significance in our analysis; and rs1889680, tagging the *CDKN2A/MTAP* region on chromosome 9 which reached suggestive levels of significance (*P* = 3.0 x 10^−7^) in both analyses (S4 Fig) [21]. The association of *HERC2/OCA2* and *CKDN2A/MTAP* loci with melanoma risk were confirmed in subsequent meta-analyses [25, 40].

### Heritability analysis of melanoma

To estimate the narrow-sense heritability (h^2^) of melanoma, we performed further QC and used the REML approach found in GCTA in 1,888 cases and 990 controls. After adjustment for imperfect LD between causal and genotyped SNPs and correction for ascertainment bias assuming a population prevalence (K) of 2% (https://seer.cancer.gov), we estimated the heritability of melanoma to be 0.18 (*P* = 0.03), closely approximating the previously published value of 0.19, and indicating that genetics contributes significantly to melanoma etiology [30].

### Heritability explained by known melanoma variants

To determine the proportion of the heritability accounted for by known melanoma-associated variants, we used the 39 SNPs in the NHGRI catalog with association P-value < 10^−5^ (S1 Table). Six of the 39 SNPs were excluded from our analysis since they were not genotyped directly and there was no LD information regarding these SNPs in either HapMap or 1000 Genomes Project databases. We found that the remaining 33 SNPs explained about 4% (95% CI = 0.02–0.06) of the variability in melanoma and 22% of the heritability explained by all common SNPs, both known and unknown. As the majority of these SNPs are likely tagging SNPs for causal variants located in their vicinity, we expanded our analysis to include SNPs located within 250 kb (upstream and downstream) of the 39 SNPs. Heritability attributable to these GWAS regions was estimated to be 0.05 (95% CI = 0.03–0.07), indicating that most of the heritability of melanoma could not be explained by already-identified variants.

### Partitioning of heritability by chromosome

It is generally assumed that common variants contribute to complex diseases under a polygenic model, in which a large number of variants distributed throughout the genome each contribute only a small amount to disease risk. A prediction of this model is that heritability should be proportional to chromosome size. Therefore, we estimated the heritability explained by each chromosome (S2 Table), and investigated the strength of the linear correlation between chromosome’s physical length and heritability, using linear regression. We did not observe a linear relationship between chromosome length and heritability (r = 0.08, *P* = 0.71).

**Fig 1:**
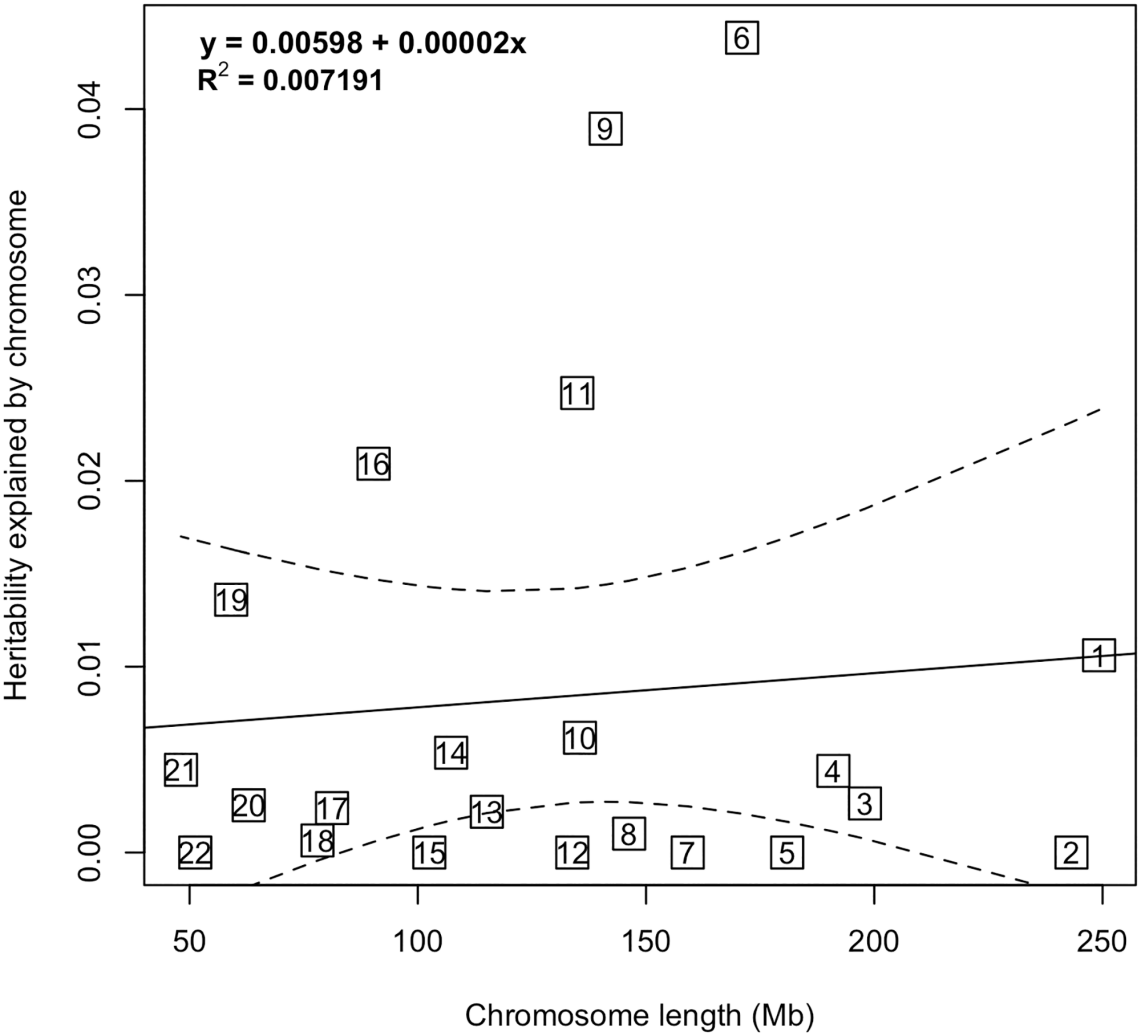
Melanoma heritability stratified by chromosome. Heritability estimates for each chromosome are plotted against chromosome length. The solid line is the regression of heritability on chromosome length and the dashed lines represent the 95% confidence intervals for the slope of the regression line. Chromosomes 6, 9, 11 and 16 are outside the 95% confidence interval and have higher heritability estimates than that expected based on chromosome length.

Chromosomes 6, 9, 11, 16 fell outside the 95% confidence bounds for the regression line, suggesting either that there is an excess of risk variants on these chromosomes, or that the risk variants on these chromosomes contribute disproportionately to melanoma risk (Fig 1). Chromosomes 9 and 16 harbor the known melanoma susceptibility loci, *CDKN2A* and *MC1R*, respectively. Therefore, we conditioned on the most significant SNP in each locus and re-ran the heritability analysis. Chromosome 9’s heritability decreased from 0.04 to 0.02 after fitting the most significant SNP at chromosome 9 (rs2383202), suggesting the existence of other risk loci on Chromosome 9. When we removed the influence of rs4408545, the most significantly associated SNP on chromosome 16, the estimated heritability for this chromosome fell from 0.02 to 0.008, suggesting that virtually all of the heritability attributable to this chromosome was due to the *MC1R* locus.

For chromosome 6, we hypothesized that the human leukocyte antigen (HLA) region on chromosome 6, which contains essential immune system genes, may be responsible for the high heritability attributed to chromosome 6. After excluding the HLA region from the heritability analysis, the heritability estimate fell by half, from 0.04 to 0.02. For chromosome 11, although there are multiple studies pointing to the existence of melanoma tumor-suppressor genes on this chromosome [41–43], no candidate gene has been identified. Consequently, we were not able to explore the contribution of this chromosome to the heritability of melanoma heritability further.

To determine whether risk SNPs throughout the remainder of the genome fit a polygenic model, we removed HLA and conditioned on rs2383202 and rs4408545 and again found there was not a significant correlation between chromosome length and heritability (r = 0.18, *P* = 0.42). Even when we removed chromosomes 6, 9, 11, and 16 completely from the analysis, there was no correlation (r = -0.01, *P* = 0.96). Taken together, these data could mean that the contribution of common variants to melanoma susceptibility is not consistent with a polygenic model. Alternatively, it is also possible that the lack of correlation between chromosome length and heritability is due to the large error associated with each heritability estimate when performed on a per chromosome basis.

### Partitioning of heritability by MAF and rare variants model

In order to characterize the allelic frequency spectrum of the genomic variation contributing to melanoma risk and test whether rare variants play a disproportionate role in melanoma risk, we partitioned the SNPs into MAF bins and estimated the heritability to attributable to each bin (S5 Fig, S3 Table). There were no significant differences in heritability estimates between bins.

Additionally, to explore the contribution of rare variants to melanoma heritability, we adjusted the heritability estimate under a rare variants model using MAF ≤ 0.01 as proxies for causal variants. The heritability of melanoma under the rare variants model was estimated to be 0.25 (SE = 0.13, *P* = 0.03), not significantly different than our initial heritability estimate.

### Partitioning of heritability by function annotation

We partitioned the heritability of melanoma by functional annotation (genic or skin *cis*-eQTLs) to determine if variants in either category explain a greater proportion of heritability than would be expected based on their frequency on the GWAS array. While genic SNPs comprised 49% of all interrogated SNPs, they accounted for 67% of the total melanoma heritability (h^2^ = 0.11, SE = 0.07; Table 1).

**Table 1:** Heritability of melanoma partitioned based on genic annotation.

Partition	Number of SNPs	% of Total SNPs	Heritability (SE)	% of Total Heritability
**Genic**	369,588	49%	0.11 (0.07)	67%
**Intergenic**	386,414	51%	0.06 (0.08)	33%

The number of SNPs (proportion of total SNPs), heritability (proportion of total heritability) and standard errors are listed for each partition.

Skin *cis*-eQTLs also accounted for a greater proportion of heritability than expected. As eQTL annotation was available for only 68% of all genotyped SNPs (N = 514,445), we first calculated the heritability attributable to this subset of variants (h^2^ = 0.11, SE = 0.08). When using eQTL p-value thresholds of 0.01, 0.001, and 0.0001, approximately 23%, 10%, and 6% of all genotyped SNPs were considered *ci*s-eQTLs (S4 Table). When estimating heritability using only these eQTLs, we found that they accounted for 54% (h^2^ = 0.06, SE = 0.05), 31% (h^2^ = 0.03, se = 0.03) and 26% (h^2^ = 0.03, SE = 0.02), respectively, of the total heritability attributable to variants for which eQTL annotation was available.

### The effect of skin color on melanoma heritability

As skin color is a modifier of biologically effective UVR exposure and melanoma susceptibility, we hypothesized that by accounting for skin color in the GWAS, we could reduce non-genetic heterogeneity and improve power to detect associations. We investigated this by performing the GWAS separately in light-skinned (skin color 1–3; 613 cases and 442 controls) and dark-skinned (skin color 4–10; 402 cases and 548 controls) whites but detected no chromosomal regions that reached genome-wide significance in either subset, likely because skin color was unavailable for significant number of cases, thereby reducing significantly the power of each skin-color-specific GWAS (data not shown).

**Fig 2:**
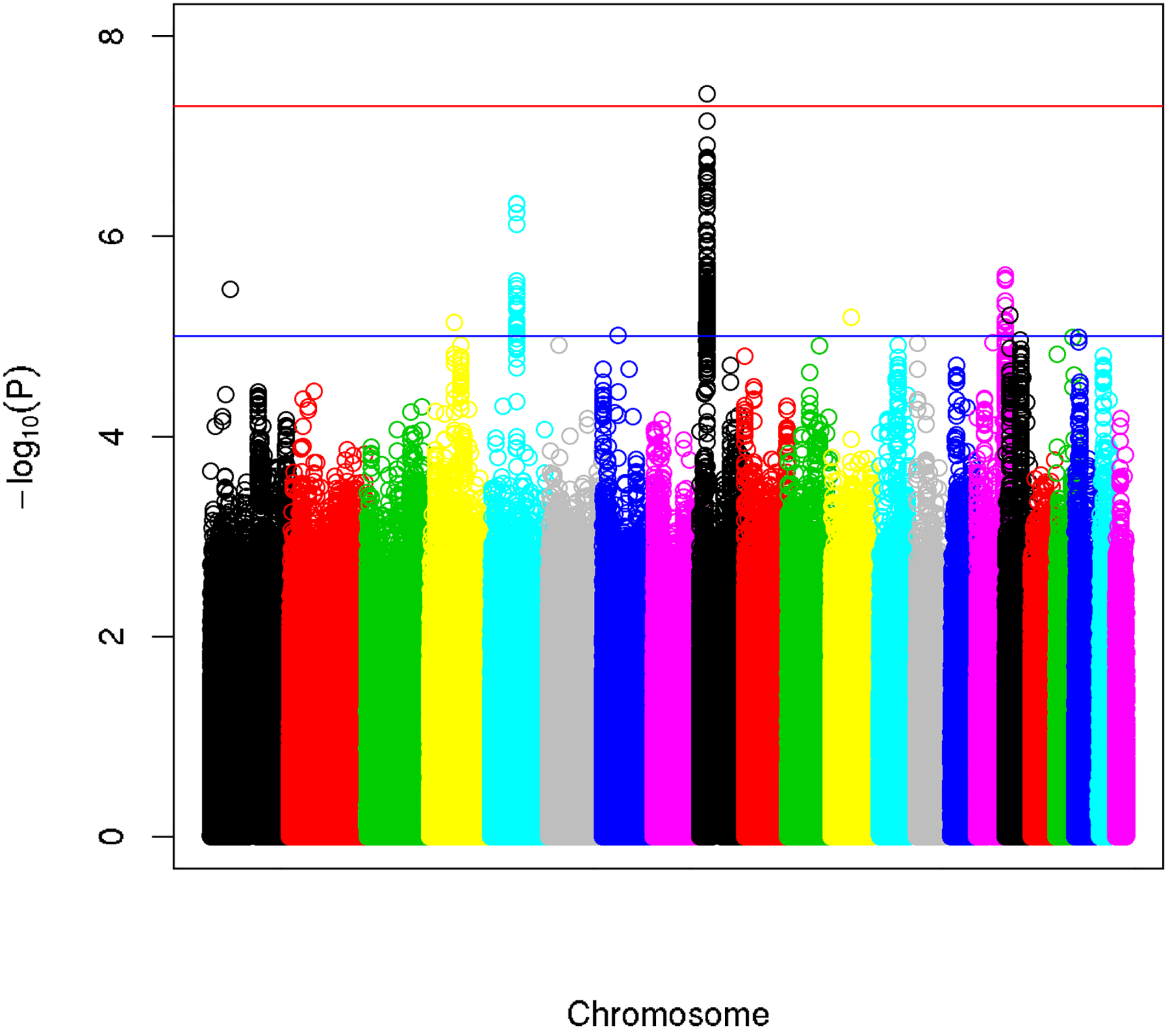
The *CDKN2A* region surpasses the genome-wide significance threshold whereas the *MC1R* and *HERC2/OCA2* loci do not upon conditioning on skin color in the melanoma etiology GWAS. Manhattan plot of the p-values for the association between imputed SNPs and melanoma using skin color as a covariate. The x-axis shows the chromosomal positions whereas the y-axis shows the–log_10_ p-values of the SNPs. The p-values were obtained by logistic regression analysis including age, sex, skin color and the first two PCs from the PCA of GWAS (S3 Fig) as covariates. The red horizontal line is the widely used genome-wide significance threshold (p = 5 x 10^−8^) that was estimated by correcting independent common variants, which is roughly 1,000,000. The blue horizontal line is the suggestive significance threshold (p = 1 x 10^−5^). The *CDKN2A* region on chromosome 9 (black) reaches genome-wide significance upon the inclusion of skin color as a covariate in the analysis while the pigmentation loci *MC1R* (chromosome 16) and *HERC2/OCA2* (chromosome 15) are no longer significant.

Next, we repeated the GWAS in all cases and controls using skin color as a covariate. In doing so, the melanoma risk locus on chromosome 9 upstream of the *MTAP/CDKN2A* region reached genome-wide significance (without skin color as a covariate: per-allele odds ratio (OR) = 1.32, *P* = 3.0 x 10^−7^; with skin color as a covariate: per-allele OR = 1.42, *P* = 3.8 x 10^−8^) (Fig 2). In contrast, the *MC1R* (chromosome 16) and *HERC2/OCA2* (chromosome 15) loci, both of which affect pigmentation, lose their significance, implying that they influence melanoma risk through their effects on pigmentation phenotypes.

### eQTL enrichment analysis

**Fig 3:**
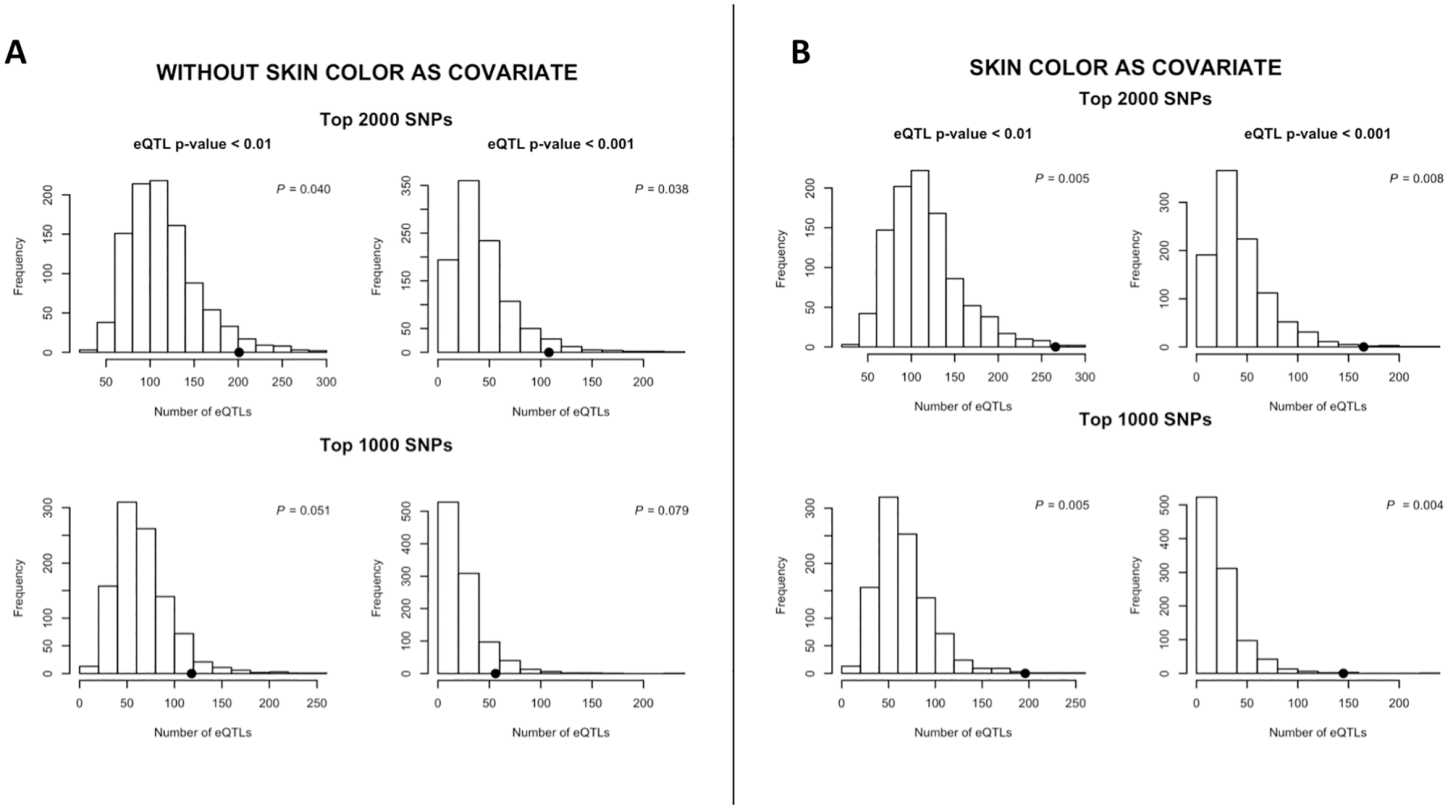
eQTL enrichment in the melanoma GWAS dataset becomes more significant upon conditioning on skin color. The distributions of the number of skin cis-eQTLs (at two different p-value thresholds: p < 0.01 and p < 0.001) in the permuted 1,000 GWAS datasets, generated by shuffling case-control status each time, are shown in the histograms. In each permuted dataset, the number of eQTLs are determined among the top 2,000 (top panel) or top 1,000 (bottom panel) associations. The solid black circle is the actual eQTL count among the melanoma-associated SNPs. The p-values correspond to empirical p-values, calculated as the proportion of permuted GWAS datasets in which the eQTL count exceeds the actual observed count among the top associations. **Panel (A)** displays results of the analysis without conditioning on skin color. Enrichment of eQTLs among the top 1,000 melanoma associated SNPs is statistically significant (*P* < 0.05), while enrichment among the top 2,000 SNPs fails to reach statistical significance but is highly suggestive. **Panel (B)** displays results of the analysis in which skin color is included as a covariate in the analysis. Upon conditioning on skin color, enrichment of eQTLs among the top melanoma-associated SNPs becomes more pronounced and is statistically significant at all thresholds tested.

Our results suggest both that functional SNPs are enriched among melanoma-associated variants, and that skin color may be an important determinant of the genetic architecture of melanoma susceptibility. If so, then we hypothesized that incorporating skin color as a covariate in our analysis should improve the power of our study, resulting in an enrichment of functional SNPs among the set of variants most associated with melanoma risk. To test this, we investigated the consequence of conditioning on skin color on the association between melanoma risk and skin *cis*-eQTLs. By permutation analysis, we found a statistically significant enrichment of skin *cis*-eQTLs among the 2,000 most significant SNPs when not conditioning on skin color at two different p-value thresholds for *cis*-eQTLs (*P*_.01_ = 0.040 and *P*_.001_ = 0.038) (Fig 3A). When skin color was included as a covariate, however, enrichment of *cis*-eQTLs among the top melanoma-associations became strikingly more significant (*P*_.01_ = 0.005, *P*_.001_ = 0.008) (Fig 3B). Results were similar when we looked only at the top 1,000 SNPs (*P*_.01_ = 0.051 and *P*_.001_ = 0.079; conditional analysis: *P*_.01_ = 0.005, *P*_.001_ = 0.004). Thus, our data demonstrate that by accounting for skin color, we improve significantly the power of our study to detect an enrichment of functional SNPs among those SNPs most associated with melanoma.

## Discussion

This goal of this study was to investigate the genetic landscape of melanoma susceptibility and dissect the contributions of genetic and environmental variation by integrating GWAS data with pigmentation and expression quantitative trait loci data. We used the GCTA software tool for our analyses, which has previously been applied to study the heritability of other complex diseases including schizophrenia [44], Tourette Syndrome, obsessive-compulsive disorder [45] and multiple myeloma [46]. Although the overall heritability of the melanoma dataset we investigated had previously been estimated to be 0.19 using GCTA [30], little is known of how the genetic contribution to risk is distributed throughout the genome. Thus, by bridging genetics and biology, our study is the first to yield insights into the component parts of the genetic architecture of melanoma risk.

We found that rare variants were not disproportionately represented among melanoma-associated variants, suggesting that post-GWAS studies relying on common variation will continue to be informative for melanoma susceptibility. We then demonstrated that a large proportion of the heritability of melanoma remains unknown. The heritability explained by known GWAS SNPs for melanoma risk (*h*^*2*^ = 0.04) or the regions they reside in (including SNPs within 250 kb of the GWAS SNPs; *h*^*2*^ = 0.05) was only a small fraction of the heritability explained by all common SNPs (*h*^*2*^ = 0.18), suggesting that more common melanoma risk loci are yet to be discovered.

Having shown the significant contribution of common variants to the heritability of melanoma, we went on to examine their genomic distribution by partitioning the heritability by chromosome. We found that melanoma susceptibility is consistent with an oligogenic model, in which a few genomic regions having relatively large effects, rather than many risk loci of very small effect sizes distributed uniformly throughout the genome, as is characteristic of highly polygenic traits [47].

We found that the high heritabilities of chromosomes 9 and 16, which explain a significantly greater proportion of the variance than expected based on their size, were mostly attributable to two regions of known association: the *CDKN2A/MTAP* region on chromosome 9 and *MC1R* region on chromosome 16. Chromosome 6 explained the largest amount of heritability, and its contribution was mostly attributable to the HLA locus which contains a large number of immune-related genes [48]. It is possible that certain HLA haplotypes are associated with melanoma risk by virtue of allowing early stage melanoma to evade the immune system. In fact, loss of class I expression has been frequently documented as an immune escape mechanism in melanomas which would go along with this hypothesis [49]. Although there are currently no known GWAS variants associated with melanoma in the HLA region, it is possible that previous GWAS were underpowered to detect these associations. One possible explanation is that there are many variants with small effect sizes within the HLA locus, such that although the overall contribution of the HLA locus to melanoma heritability is high, larger studies with higher power are needed to identify individual variants within the locus Our finding that much of melanoma heritability is explained by HLA may be of particular interest in light of the fact that the use of immunotherapy to treat cancer was pioneered in melanoma. By stratifying patients into immune responders vs. non-responders, it may be possible to identify biomarkers in HLA that can be used clinically to aid in treatment decision-making for melanoma and other cancer patients.

Rare or low-frequency variants that are poorly captured by genotyping arrays are another potential source of missing heritability [50]. We investigated the hypothesis that melanoma heritability is mostly attributable to rare variants by partitioning the heritability by MAF as well as estimating the heritability under the assumption that causal variants have MAF ≤ 0.01. However, we found no significant differences between the heritabilities captured by SNPs in different MAF bins and no substantial increase in the heritability estimate under the rare variants model. Taken together, our data provides no support for the hypothesis that rare or low-frequency variants contribute substantially to missing heritability.

The majority of variants associated with complex diseases are in non-coding regions and are thought to act by influencing gene expression [51–53]. Hence, variants identified by GWAS as reproducibly associated with a number of complex traits are enriched for eQTLs [51, 54–57]. Consistent with this observation, we detected an enrichment of skin *cis*-eQTLs among top associations in melanoma, providing strong evidence that the genetic regulation of gene expression in the skin is important in melanoma susceptibility. This has important implications for GWAS, as gene expression in the biologically relevant tissue is a potentially powerful intermediate phenotype that can help the functional characterization of GWAS variants.

Finally, by incorporating skin color, a known risk factor for melanoma, in our analysis of a European-only population, we significantly potentiated the enrichment of eQTLs among melanoma susceptibility loci. We also uncovered a significant association in the *CDKN2A/MTAP* region, previously reported in two separate GWAS of melanoma in Caucasians [19, 23]. This region contains the *CDKN2A* gene, which has a well-characterized role in cell cycle regulation [58, 59] and is responsible for a substantial proportion of familial melanomas [60, 61]. Furthermore, we found that the *MC1R* and *HERC2/OCA2* loci were no longer significant when conditioning on skin color. Several studies have previously shown the presence of variants within the *MC1R* locus that may have effects on melanoma risk independent of their effect on pigmentation [62, 63]. Although none of the MC1R variants remained significant after conditioning on skin color in our study, it is possible that our study was underpowered to detect the pigmentation-independent effects.

Our finding that the *CDKN2A/MTAP* region becomes significant after accounting for skin color in the model suggests that the genetics underlying melanoma risk may differ between light- and dark-skinned Caucasian populations such that the inclusion of skin color in the model allows the discovery of loci which might affect melanoma risk differently in the two subsets. Genetic studies comparing melanoma susceptibility in Northern European and darker-skinned Greek populations provide support for this hypothesis. One study reported that 15% of all melanoma cases in Greece have germline mutations in *CDKN2A* [64]. This was much higher than the prevalence of mutations in this gene in an Australian population of Northern European ancestry (0.2%) [65], suggesting that the genetic architecture of melanoma susceptibility may differ between light- and dark-skinned Caucasian populations. The hypothesis that differences in skin color may represent underlying population structure with different genetic architectures that is unaccounted for by solely pigmentation phenotypes, would also explain why the effect of the variation in pigmentation genes does not similarly affect the penetrance of the *CDKN2A/MTAP* region.

A limitation our study is that the skin color data was self-reported based on a questionnaire. However, when we ran the GWAS using self-reported skin color as the phenotype, we found that the most significant loci were rs4268748 on chromosome 16 near *MC1R* (*P* = 1.76 x 10^−14^), rs12913832 on chromosome 15 near *OCA2* (*P* = 6.08 x 10^−11^) and rs12203592 on chromosome 6 near *IRF4* (*P* = 5.79 x 10^−9^), which have all been previously shown to be associated with skin color in Europeans [66, 67]. This suggests that self-reported skin color is in fact a reliable estimator and that the potential bias introduced into the study by using this subjective measure, should be minimal. A second limitation of our study is that skin color was only available for approximately half of the cases and all of the controls. This further reduced our sample sizes and thus power, when we stratified the dataset into subsets based on skin color, running the analyses separately in each subset. Another limitation of our study is that although skin color is an important modifier of melanoma risk, there are other factors such as number of nevi [68] and sun exposure [69, 70] that have also shown to be important modifiers of melanoma risk. Inclusion of these parameters in the model would allow us to investigate the full spectrum of environmental contributions to melanoma risk, on the other hand, this would add additional layers of subjectivity and complexity making the data harder to interpret.

Our conclusion that there may be differences in genetic susceptibility to melanoma risk between light- and dark-skinned individuals only applies to Caucasian populations as melanoma in individuals of African, Hispanic and Asian ancestry may have a different, perhaps not UV-related, etiology in these populations, often appearing in sun-protected atypical locations such as acral, subungual and mucosal skin [71, 72]. Although for melanoma, there appears to be an established relationship between skin color and melanoma risk, for most other complex diseases, the effect of environment on phenotype is too complex to be accounted for. This is further compounded by the fact that it is hard to objectively quantify most environmental exposures, further limiting the applicability of our approach to the study of other complex traits.

The next step in advancing our understanding of melanoma risk and heritability would be to perform the GWAS and heritability analyses separately in light- and dark-skinned individuals. We attempted these analyses with our dataset, however our sample size was too small to reveal any significant hits or yield reliable heritability estimates in the light- and dark-skinned subsets. These analyses should be attempted in a larger cohort to see if the heritability of melanoma differs between light- and dark-skinned Caucasians and to identify variants that are specific to each skin color group.

Taken together, these results have important implications for future studies of melanoma susceptibility, as they suggest that skin color should be accounted for as a covariate and that the genetic architecture of melanoma risk differs between light-skinned and dark skinned whites. More broadly, our strategy of integrating known non-genetic risk factors such as skin color into heritability analyses may have important implications for genomic investigations into the genetic architecture of other complex diseases as well. We speculate that this approach may facilitate the identification of patient subsets in whom the biology of risk differs significantly, thereby providing a more nuanced understanding of the role of genetics in human disease.

## Supporting information

S1 Fig PCA plot of the melanoma GWAS dataset. PCA plot of the melanoma GWAS samples along with HapMap reference populations of Northern European (CEU), African (YRI) and Asian (ASN = CHB + JPT) ancestry. The samples used in PCA are color-coded as indicated in the legend on the right. While most GWAS samples cluster tightly with the HapMap CEU population, a small number tend toward YRI or ASN, indicating small amounts of non-European ancestry. The individuals who fall outside the cluster boundaries, as indicated by the black circle, were defined population outliers.
(TIF)

S2 Fig PCA plot of the melanoma GWAS samples showing within Europe ancestry. PCA plot of the melanoma GWAS samples along with reference populations of Northern European (CEU), Ashkenazi Jewish (AJ) and Tuscan (TSI) ancestry. GWAS are color-coded according to case-control status (A), and skin color (B), as indicated in the legend for each panel.
(TIF)

S3 Fig PCA plot of the melanoma GWAS samples after removal of population outliers. PCA plot of the melanoma GWAS samples after the removal of population outliers with non-European ancestry (individuals that are outside the black circle in S1 Fig). Cases and controls are color-coded as indicated in the legend on the right. There are no significant differences in PC1 or PC2 between cases and controls. The first two PCs from the plot are used as covariates in the association analyses of melanoma to correct for population structure.
(TIF)

S4 Fig Manhattan plot of the melanoma GWAS. Manhattan plot of the p-values for the association between imputed SNPs and melanoma. The x-axis shows the chromosomal positions whereas the y-axis shows the–log_10_ p-values of the SNPs. The p-values were obtained by logistic regression analysis including age, sex and the first two PCs from the PCA of GWAS as covariates (S3 Fig). The red horizontal line is the widely used genome-wide significance threshold (p = 5 x 10^−8^) that was estimated by correcting independent common variants, which is roughly 1,000,000. The blue line is the suggestive significance threshold (p = 1 x 10^−5^). The *MC1R* region on chromosome 16 (magenta) is significantly associated with melanoma risk, whereas the *CDKN2A* region on chromosome 9 (black) and the *HERC2/OCA2* region on (dark blue) chromosome 15 reach suggestive significance.
(TIF)

S5 Fig Melanoma heritability partitioned by minor allele frequency. The x-axis represents the MAF bins while the y-axis represents the heritability attributed to SNPs in the corresponding MAF bins. The standard errors of the heritability estimates are represented by the error bars.
(TIF)

S1 Table Melanoma susceptibility SNPs from the NHGRI GWAS catalog. Chromosomal regions, reported genes, risk allele frequencies, odds ratios (OR) or beta-coefficients and study references are listed for all melanoma-associated SNPs downloaded from the NHGRI GWAS Catalog.
(DOCX)

S2 Table Heritability of melanoma partitioned by chromosome. Heritability estimates and standard errors (SE) are listed for each chromosome.
(DOCX)

S3 Table Heritability of melanoma partitioned by minor allele frequency (MAF). The number of SNPs (proportion of total SNPs), heritability (proportion of total heritability) and standard errors (SE) are listed for SNPs in each MAF bin.
(DOCX)

S4 Table Heritability of melanoma partitioned based on skin *cis*-eQTL annotation at three different p-value thresholds. The number of SNPs (proportion of total SNPs), heritability (proportion of total heritability) and standard errors (SE) are listed for each partition.
(DOCX)
