# Supplementary figures and images for: Integrative genetic analysis suggests that skin color modifies the genetic architecture of melanoma
Source: PLoS One. 2017 Oct 3;12(10):e0185730. doi: 10.1371/journal.pone.0185730 (PMC5626488; doi:10.1371/journal.pone.0185730)

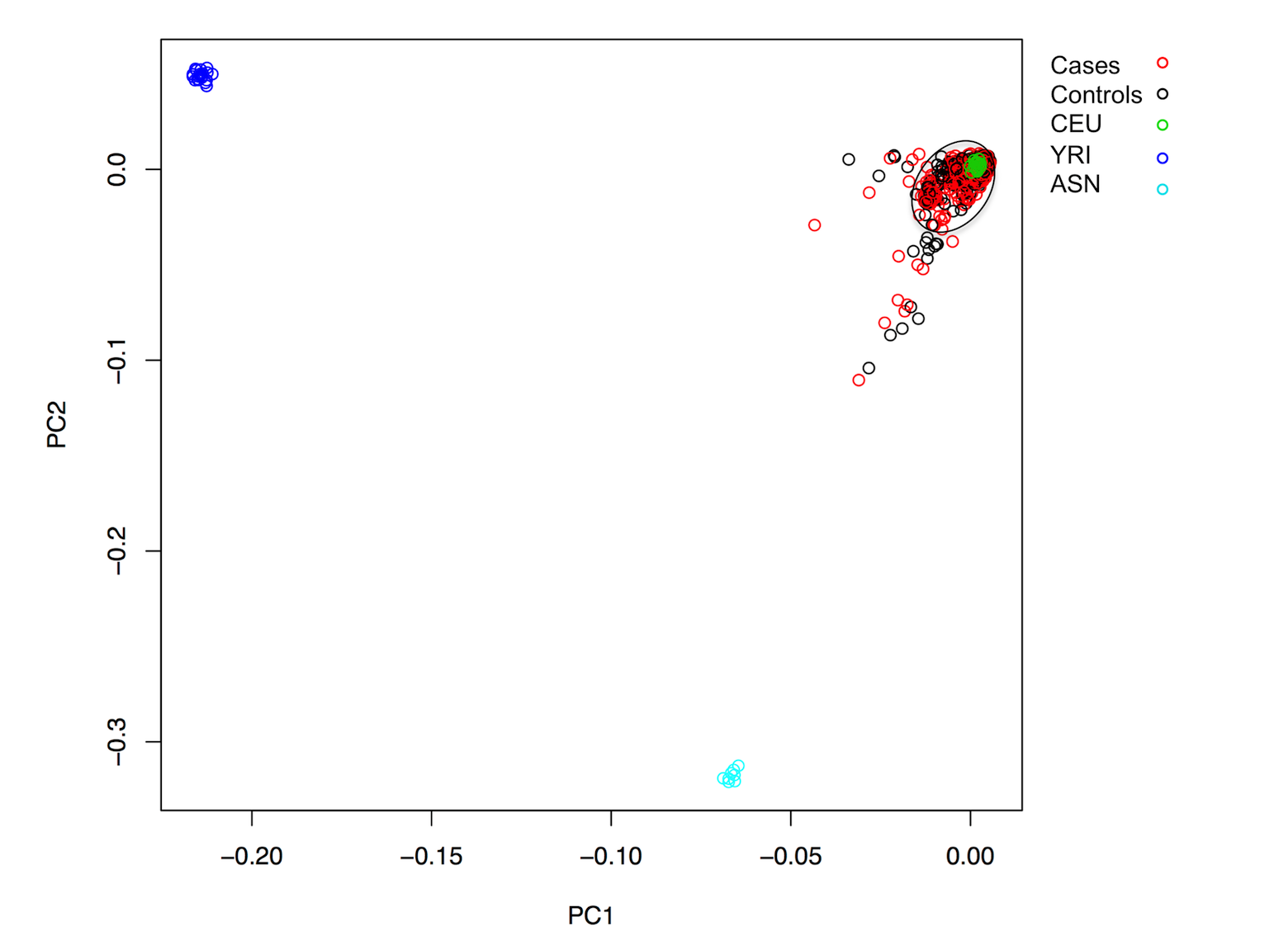

Supplement: S1 Fig — PCA plot of the melanoma GWAS samples along with HapMap reference populations of Northern European (CEU), African (YRI) and Asian (ASN = CHB + JPT) ancestry. The samples used in PCA are color-coded as indicated in the legend on the right. While most GWAS samples cluster tightly with the HapMap CEU population, a small number tend toward YRI or ASN, indicating small amounts of non-European ancestry. The individuals who fall outside the cluster boundaries, as indicated by the black circle, were defined population outliers. (TIF) [file pone.0185730.s001.tif]

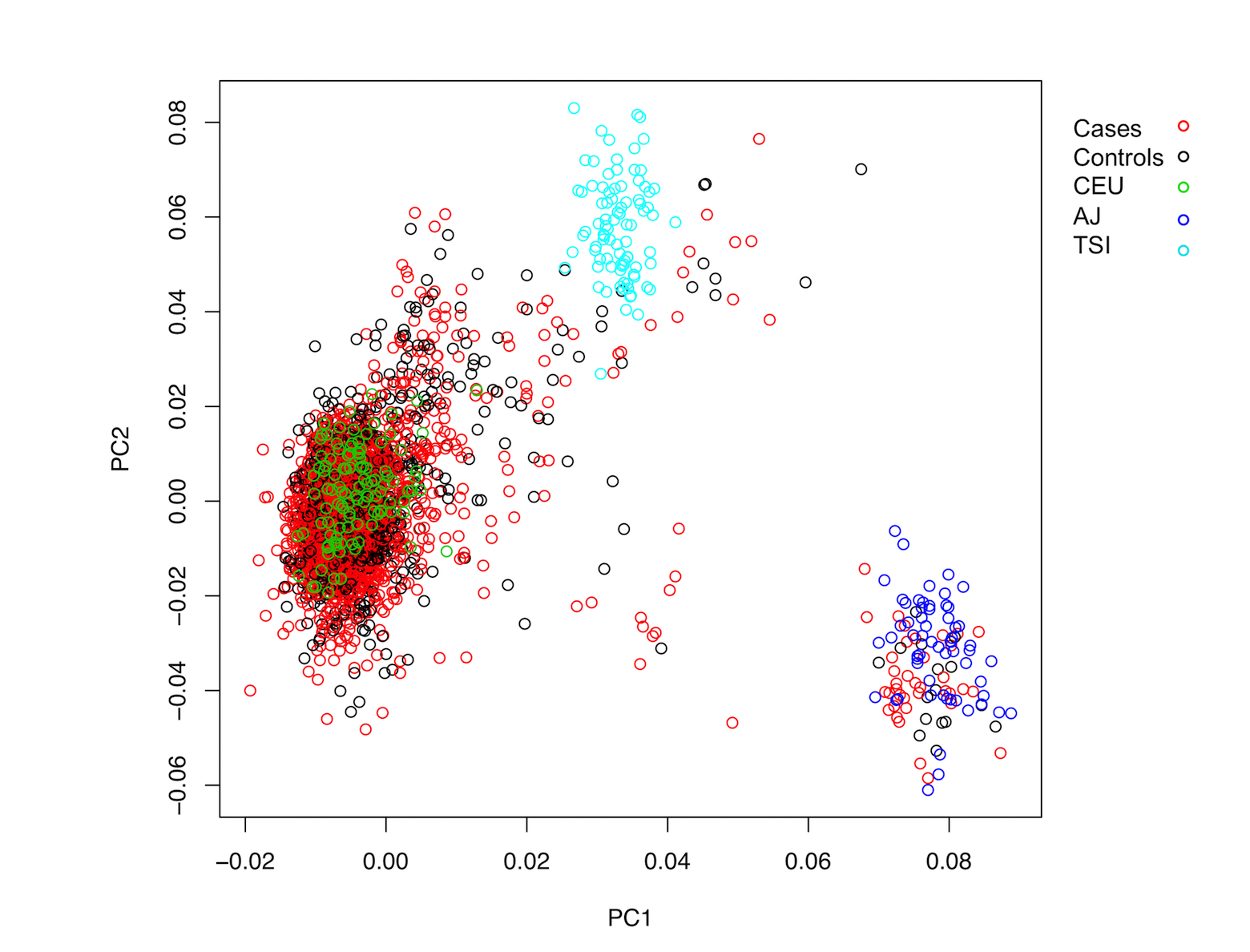

Supplement: S2 Fig — PCA plot of the melanoma GWAS samples along with reference populations of Northern European (CEU), Ashkenazi Jewish (AJ) and Tuscan (TSI) ancestry. GWAS are color-coded according to case-control status (A), and skin color (B), as indicated in the legend for each panel. (TIF) [file pone.0185730.s002.tif]

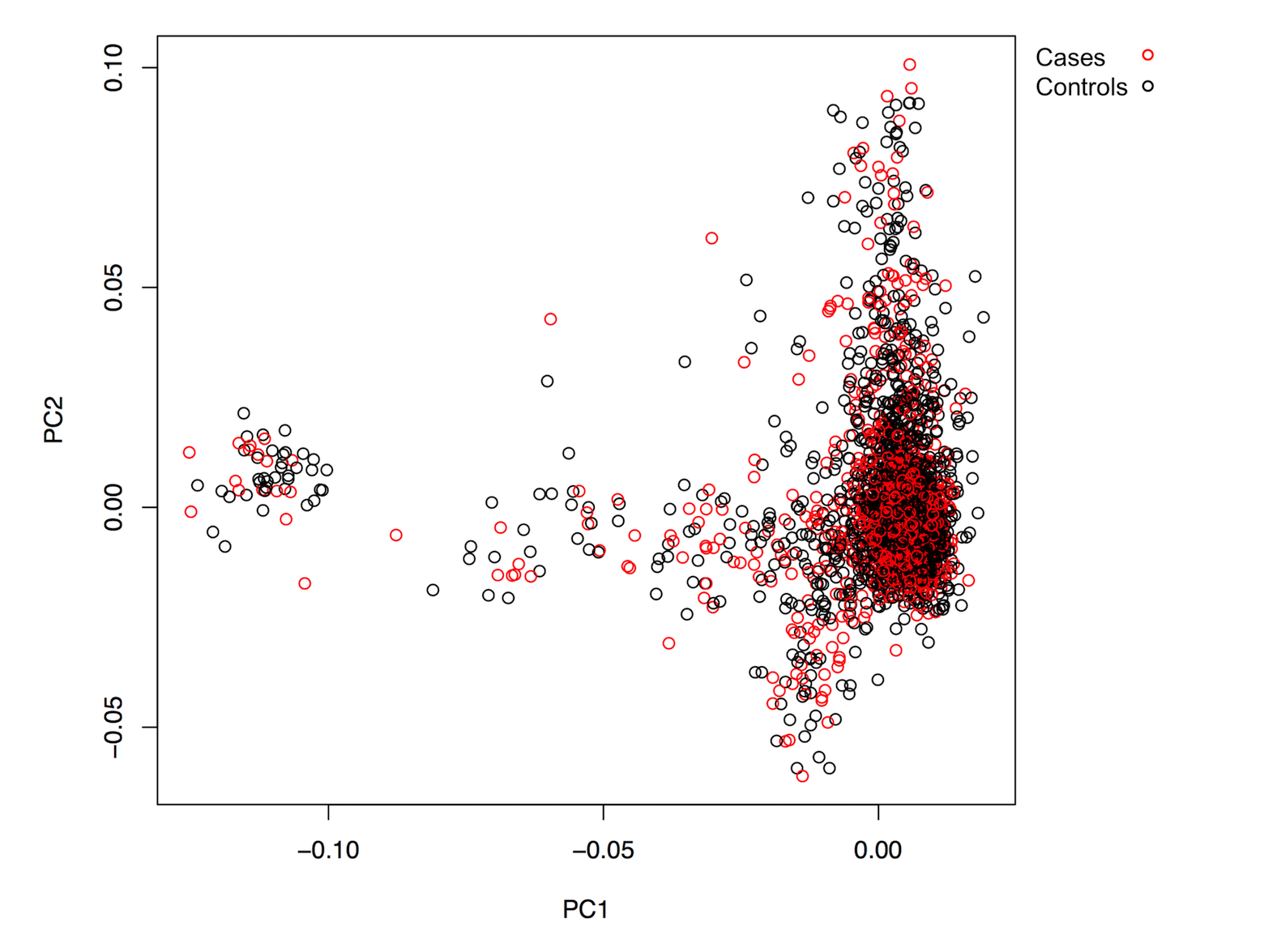

Supplement: S3 Fig — PCA plot of the melanoma GWAS samples after the removal of population outliers with non-European ancestry (individuals that are outside the black circle in S1 Fig). Cases and controls are color-coded as indicated in the legend on the right. There are no significant differences in PC1 or PC2 between cases and controls. The first two PCs from the plot are used as covariates in the association analyses of melanoma to correct for population structure. (TIF) [file pone.0185730.s003.tif]

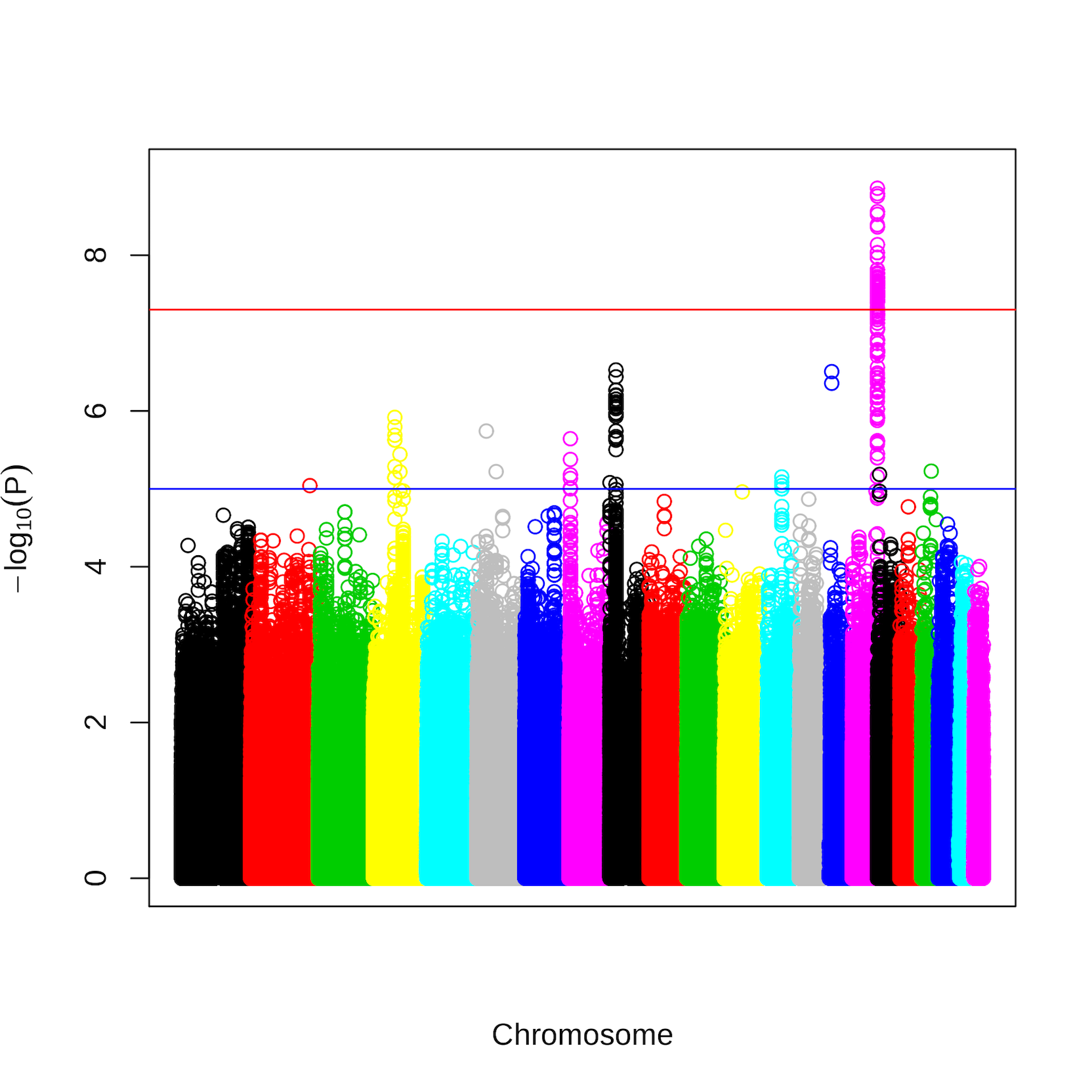

Supplement: S4 Fig — Manhattan plot of the p-values for the association between imputed SNPs and melanoma. The x-axis shows the chromosomal positions whereas the y-axis shows the–log10 p-values of the SNPs. The p-values were obtained by logistic regression analysis including age, sex and the first two PCs from the PCA of GWAS as covariates (S3 Fig). The red horizontal line is the widely used genome-wide significance threshold (p = 5 x 10^−8^) that was estimated by correcting independent common variants, which is roughly 1,000,000. The blue line is the suggestive significance threshold (p = 1 x 10^−5^). The MC1R region on chromosome 16 (magenta) is significantly associated with melanoma risk, whereas the CDKN2A region on chromosome 9 (black) and the HERC2/OCA2 region on (dark blue) chromosome 15 reach suggestive significance. (TIF) [file pone.0185730.s004.tif]

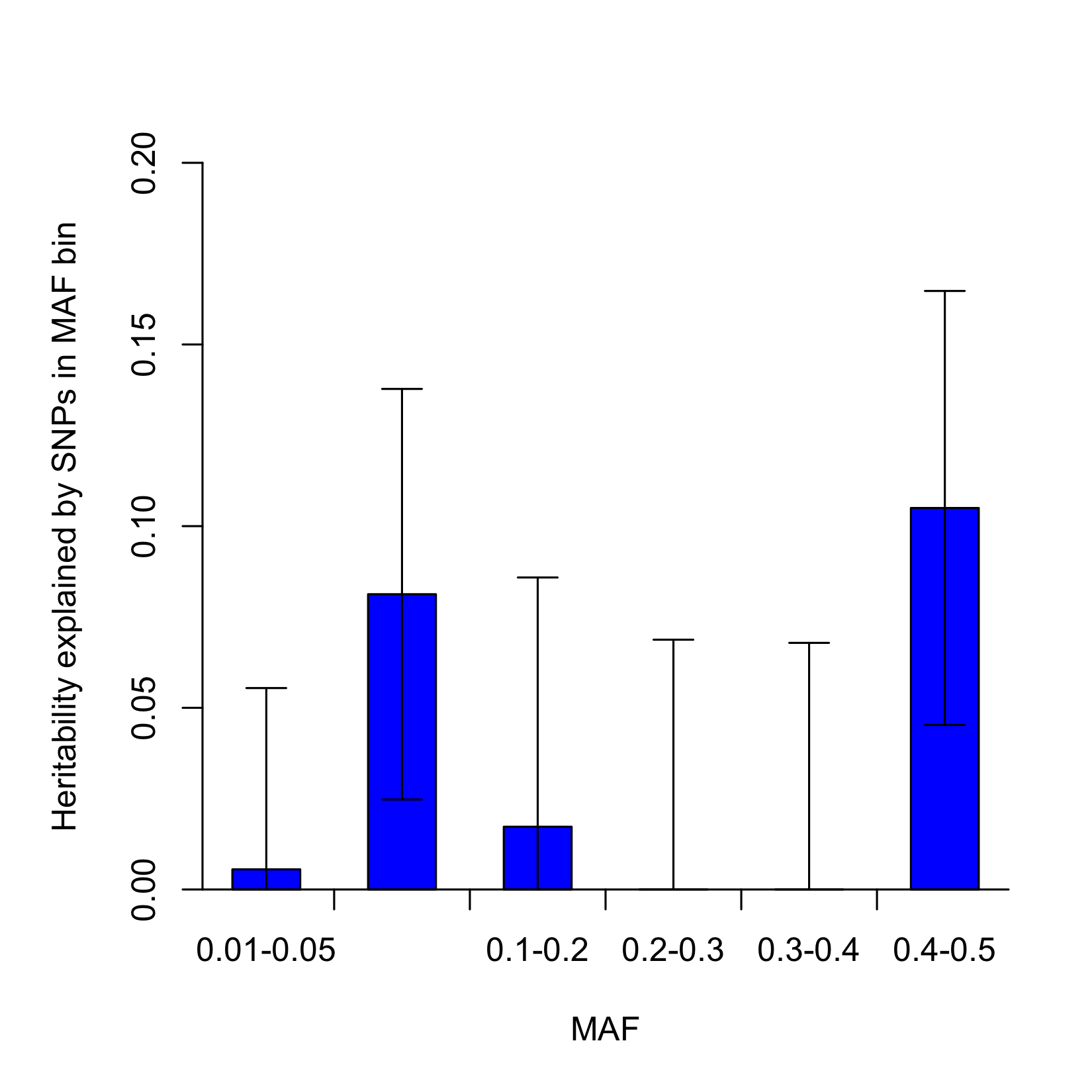

Supplement: S5 Fig — The x-axis represents the MAF bins while the y-axis represents the heritability attributed to SNPs in the corresponding MAF bins. The standard errors of the heritability estimates are represented by the error bars. (TIF) [file pone.0185730.s005.tif]
