# Supplementary material for: Integrative genetic analysis suggests that skin color modifies the genetic architecture of melanoma
Source: PLoS One. 2017 Oct 3;12(10):e0185730. doi: 10.1371/journal.pone.0185730 (PMC5626488; doi:10.1371/journal.pone.0185730)
Supplement: S1 Table — Chromosomal regions, reported genes, risk allele frequencies, odds ratios (OR) or beta-coefficients and study references are listed for all melanoma-associated SNPs downloaded from the NHGRI GWAS Catalog. (DOCX) [file pone.0185730.s006.docx]

**S1 Table. Melanoma susceptibility SNPs from the NHGRI GWAS Catalog.** Chromosomal regions, reported genes, risk allele frequencies, odds ratios (OR) or beta-coefficients and study references are listed for all melanoma-associated SNPs downloaded from the NHGRI GWAS Catalog.

| **Region** | **Reported Genes** | **SNP** | **Risk Allele Frequency** | **OR or beta** | **Reference** |
| --- | --- | --- | --- | --- | --- |
| 2p15 | *WDPCP* | rs186133190 | 0.015 | 0.44 unit increase | Law et al., 2015 |
| 2p22.2 | *CYP1B1, RMDN2* | rs6750047 | 0.43 | 0.092 unit increase | Law et al., 2015 |
| 3q26.2 | *TERC* | rs12696304 | 0.27 | 0.092 unit decrease | Law et al., 2015 |
| 6p22.3 | *CDKAL1* | rs6914598* | 0.32 | 0.1 unit increase | Law et al., 2015 |
| 7p21.1 | *AGR3* | rs1636744 | 0.40 | 0.091 unit increase | Law et al., 2015 |
| 7q31.33 | *GPR37, POT1* | rs4731207 | 0.5 | 0.079 unit increase | Law et al., 2015 |
| 7q32.3 | *LINC-PINT* | rs4731742* | 0.3 | 0.12 unit decrease | Law et al., 2015 |
| 9q31.2 | *TMEM38B, RAD23B, TAL2, ZNF462, KLF4* | rs10739221 | 0.24 | 0.12 unit increase | Law et al., 2015 |
| 9q31.2 | *KLF4* | rs7041168 | 0.47 | 0.081 unit increase | Law et al., 2015 |
| 10q24.33 | *OBFC1, SH3PXD2A* | rs2995264 | 0.088 | 0.16 unit increase | Law et al., 2015 |
| 11q13.3 | *TPCN2, CCND1* | rs2290419 | 0.044 | 0.18 unit decrease | Law et al., 2015 |
| 11q13.3 | *CCND1* | rs498136 | 0.32 | 0.12 unit increase | Law et al., 2015 |
| 12p13.1 | *GPRC5A* | rs2111398* | 0.43 | 0.087 unit increase | Law et al., 2015 |
| 15q13.1 | *OCA2* | rs4778138 | 0.16 | 0.17 unit decrease | Law et al., 2015 |
| 16q22.1 | *CDH1* | rs35158985* | 0.3 | 0.095 unit increase | Law et al., 2015 |
| 20q13.3 | *RTEL1* | rs75691080* | 0.095 | 0.16 unit decrease | Law et al., 2015 |
| 3p21.2 | *DOCK3* | rs1031925 | N/A | 1.15 | Song et al., 2014 |
| 3q26.2 | *ACTRT3* | rs13097028 | N/A | 1.1236 | Song et al., 2014 |
| 4q24 | *TET2* | rs4698934 | N/A | 1.18 | Song et al., 2014 |
| 6q12 | *EYS* | rs1889497 | N/A | 1.1494 | Song et al., 2014 |
| 11q14.3 | *TYR* | rs1847134 | N/A | 1.25 | Song et al., 2014 |
| 16q12.2 | *FTO* | rs16953002 | 0.17 | 1.16 | Iles et al., 2013 |
| 2q33.1 | *CASP8* | rs13016963 | 0.37 | 1.14 | Barrett et al., 2011 |
| 11q22.3 | *ATM* | rs1801516 | 0.87 | 1.19 | Barrett et al., 2011 |
| 21q22.3 | *MX2* | rs45430 | 0.61 | 1.14 | Barrett et al., 2011 |
| 5p15.33 | *TERT, CLPTM1L* | rs401681 | 0.46 | 1.2 | Barrett et al., 2011 |
| 5p13.2 | *SLC45A2* | rs35390* | 0.98 | 2.78 | Barrett et al., 2011 |
| 6q23.2 | *ASIP* | rs228437 | 0.33 | 1.21 | Barrett et al., 2011 |
| 22q13.1 | *PLA2G6* | rs6001027 | 0.65 | 1.18 | Barrett et al., 2011 |
| 1q21.3 | *ARNT, SETDB1, LASS2, ANXA9, MCL1, CTSK* | rs7412746 | 0.55 | 1.15 | Macgregor et al., 2011 |
| 1q42.12 | *PARP1* | rs3219090 | 0.67 | 1.15 | Macgregor et al., 2011 |
| 1q21.3 | *ANXA9* | rs1722784 | N/A | 1.12 | Amos et al., 2011 |
| 10q25.1 | intergenic | rs17119461 | 0.04 | 8.4 | Teerlink et al., 2011 |
| 9p21.3 | *CDKN2A* | rs7023329 | 0.50 | 1.18 | Bishop et al., 2009 |
| 11q14.3 | *TYR* | rs1393350 | 0.27 | 1.29 | Bishop et al., 2009 |
| 16q24.3 | *MC1R* | rs258322 | 0.09 | 1.67 | Bishop et al., 2009 |
| 16q24.3 | *MC1R* | rs4785763 | 0.32 | 1.36 | Bishop et al., 2009 |
| 22q13.1 | intergenic | rs2284063 | 0.37 | 1.2 | Bishop et al., 2009 |
| 20q11.22 | *CDC91L1* | rs910873 | 0.09 | 1.75 | Brown et al., 2008 |

* This SNP was not included in the heritability estimate explained by known GWAS SNPs since it was not genotyped or imputed in our study, and there was no LD information regarding this SNP in either the HapMap or 1000 Genomes Project databases. However, the chromosomal region spanning this SNP (250 kb up or downstream) was included in the heritability estimate explained by known GWAS regions.
