# Supplementary material for: Integrative genetic analysis suggests that skin color modifies the genetic architecture of melanoma
Source: PLoS One. 2017 Oct 3;12(10):e0185730. doi: 10.1371/journal.pone.0185730 (PMC5626488; doi:10.1371/journal.pone.0185730)
Supplement: S2 Table — Heritability estimates and standard errors (SE) are listed for each chromosome. (DOCX) [file pone.0185730.s007.docx]

**S2 Table. Heritability of melanoma partitioned by chromosome.** Heritability estimates and standard errors (SE) are listed for each chromosome.

| **Chromosome** | **Heritability** | **SE** |
| --- | --- | --- |
| 1 | 0.02 | 0.03 |
| 2 | 0.000001 | 0.02 |
| 3 | 0.003 | 0.02 |
| 4 | 0.01 | 0.02 |
| 5 | 0.000001 | 0.02 |
| 6 | 0.05 | 0.02 |
| 7 | 0.000001 | 0.02 |
| 8 | 0.008 | 0.02 |
| 9 | 0.04 | 0.02 |
| 10 | 0.01 | 0.02 |
| 11 | 0.03 | 0.02 |
| 12 | 0.000001 | 0.02 |
| 13 | 0.000001 | 0.02 |
| 14 | 0.007 | 0.02 |
| 15 | 0.000001 | 0.02 |
| 16 | 0.03 | 0.02 |
| 17 | 0.000001 | 0.02 |
| 18 | 0.000001 | 0.02 |
| 19 | 0.01 | 0.02 |
| 20 | 0.007 | 0.01 |
| 21 | 0.007 | 0.01 |
| 22 | 0.000001 | 0.01 |
