# Supplementary material for: Integrative genetic analysis suggests that skin color modifies the genetic architecture of melanoma
Source: PLoS One. 2017 Oct 3;12(10):e0185730. doi: 10.1371/journal.pone.0185730 (PMC5626488; doi:10.1371/journal.pone.0185730)
Supplement: S3 Table — The number of SNPs (proportion of total SNPs), heritability (proportion of total heritability) and standard errors (SE) are listed for SNPs in each MAF bin. (DOCX) [file pone.0185730.s008.docx]

**S3 Table.** **Heritability of melanoma partitioned by minor allele frequency (MAF).** The number of SNPs (proportion of total SNPs), heritability (proportion of total heritability) and standard errors (SE) are listed for SNPs in each MAF bin.

| **MAF** | **Number of SNPs**  **(% of Total)** | **Heritability (SE)** | **% of Total Heritability** |
| --- | --- | --- | --- |
| 0.01-0.05 | 76,502 (10.1%) | 0.006  (0.05) | 1% |
| >0.05-0.10 | 94,367 (12.5%) | 0.08  (0.06) | 41% |
| >0.10-0.20 | 167,273 (22.1%) | 0.02  (0.07) | 11% |
| >0.20-0.30 | 147,550 (19.5%) | 0.000001  (0.07) | 0% |
| >0.30-0.40 | 137,644 (18.2%) | 0.000001  (0.07) | 0% |
| >0.40-0.50 | 132,666 (17.5%) | 0.10  (0.06) | 48% |
