# Supplementary material for: Integrative genetic analysis suggests that skin color modifies the genetic architecture of melanoma
Source: PLoS One. 2017 Oct 3;12(10):e0185730. doi: 10.1371/journal.pone.0185730 (PMC5626488; doi:10.1371/journal.pone.0185730)
Supplement: S4 Table — The number of SNPs (proportion of total SNPs), heritability (proportion of total heritability) and standard errors (SE) are listed for each partition. (DOCX) [file pone.0185730.s009.docx]

**S4 Table.** **Heritability of melanoma partitioned based on skin *cis*-eQTL annotation at three different p-value thresholds.** The number of SNPs (proportion of total SNPs), heritability (proportion of total heritability) and standard errors (SE) are listed for each partition.

| **Partition** | **Number of SNPs  (% of Total)** | **Heritability**  **(SE)** | **% of Total Heritability** |
| --- | --- | --- | --- |
| **p-value<0.01** |  |  |  |
| **eQTL** | 120,999 (23%) | 0.06  (0.05) | 54% |
| **Non-eQTL** | 393446 (77%) | 0.05 (0.08) | 46% |
| **p-value<0.001** |  |  |  |
| **eQTL** | 49,738  (10%) | 0.03  (0.03) | 31% |
| **Non-eQTL** | 464,707  (90%) | 0.07  (0.08) | 69% |
| **p-value<0.0001** |  |  |  |
| **eQTL** | 30,571 (6%) | 0.03  (0.02) | 26% |
| **Non-eQTL** | 483,874  (94%) | 0.08  (0.08) | 74% |
